# Distinct alterations in probabilistic reversal learning across at-risk mental state, first episode psychosis and persistent schizophrenia

**DOI:** 10.1038/s41598-024-68004-7

**Published:** 2024-07-30

**Authors:** J. D. Griffin, K. M. J. Diederen, J. Haarsma, I. C. Jarratt Barnham, B. R. H. Cook, E. Fernandez-Egea, S. Williamson, E. D. van Sprang, R. Gaillard, F. Vinckier, I. M. Goodyer, NSPN Consortium, Edward Bullmore, Raymond Dolan, Ian Goodyer, Peter Fonagy, Peter Jones, Samuel Chamberlain, Michael Moutoussis, Tobias Hauser, Sharon Neufeld, Rafael Romero-Garcia, Michelle St Clair, Petra Vértes, Kirstie Whitaker, Becky Inkster, Gita Prabhu, Cinly Ooi, Umar Toseeb, Barry Widmer, Junaid Bhatti, Laura Villis, Ayesha Alrumaithi, Sarah Birt, Aislinn Bowler, Kalia Cleridou, Hina Dadabhoy, Emma Davies, Ashlyn Firkins, Sian Granville, Elizabeth Harding, Alexandra Hopkins, Daniel Isaacs, Janchai King, Danae Kokorikou, Christina Maurice, Cleo McIntosh, Jessica Memarzia, Harriet Mills, Ciara O’Donnell, Sara Pantaleone, Jenny Scott, Beatrice Kiddle, Ela Polek, Pasco Fearon, John Suckling, Anne-Laura van Harmelen, Rogier Kievit, Richard Bethlehem, G. K. Murray, P. C. Fletcher

**Affiliations:** 1 Department of Psychiatry, University of Cambridge, Addenbrookes Hospital, Cambridge, CB2 0QQ UK; 2 Service Hospitalo-Universitaire, GHU Paris Psychiatrie & Neurosciences, F-75014 Paris, France; 3 Motivation, Brain & Behavior (MBB) lab, Institut du Cerveau et de la Moelle épinière (ICM), F-75013 Paris, France; 4 Cambridgeshire and Peterborough NHS Trust, Cambridge, UK; 5 Coventry and Warwickshire NHS Partnership Trust, Warwick, UK; 6 Wellcome Trust MRC Institute of Metabolic Science, University of Cambridge, Cambridge Biomedical Campus, Cambridge, CB2 0QQ UK; 7 Amsterdam University Medical Centres (UMC), Amsterdam, The Netherlands; 8 Paris Descartes University, Paris, France; 9 Université Paris Cité, F-75006 Paris, France; 10 Behavioural and Clinical Neuroscience Institute, University of Cambridge, Cambridge, UK; 11 ImmunoPsychiatry, GlaxoSmithKline Research and Development, Cambridge, UK; 12 Max Planck University College London Centre for Computational Psychiatry and Ageing Research, University College London, London, UK; 13 Wellcome Centre for Human Neuroimaging, University College London, London, UK; 14 Research Department of Clinical, Educational and Health Psychology, University College London, London, UK; 15 Medical Research Council Cognition and Brain Sciences Unit, University of Cambridge, Cambridge, UK; 16 Department of Psychosis Studies, Institute of Psychiatry, Psychology and Neuroscience, London, UK; 17 Wellcome Centre for Human Neuroimaging, Queen Square, UCL, London, UK

**Keywords:** Translational research, Human behaviour

## Abstract

We used a probabilistic reversal learning task to examine prediction error-driven belief updating in three clinical groups with psychosis or psychosis-like symptoms. *Study 1* compared people with at-risk mental state and first episode psychosis (FEP) to matched controls. *Study 2* compared people diagnosed with treatment-resistant schizophrenia (TRS) to matched controls. The design replicated our previous work showing ketamine-related perturbations in how meta-level confidence maintained behavioural policy. We applied the same computational modelling analysis here, in order to compare the pharmacological model to three groups at different stages of psychosis. Accuracy was reduced in FEP, reflecting increased tendencies to shift strategy following probabilistic errors. The TRS group also showed a greater tendency to shift choice strategies though accuracy levels were not significantly reduced. Applying the previously-used computational modelling approach, we observed that only the TRS group showed altered confidence-based modulation of responding, previously observed under ketamine administration. Overall, our behavioural findings demonstrated resemblance between clinical groups (FEP and TRS) and ketamine in terms of a reduction in stabilisation of responding in a noisy environment. The computational analysis suggested that TRS, but not FEP, replicates ketamine effects but we consider the computational findings preliminary given limitations in performance of the model.

## Introduction

Psychosis is defined as a loss of contact with reality and is characterised by hallucinations and delusions. These alterations in experience and beliefs have been explored in the context of associative learning models^1,2^ and related to the predictive processing framework^3–5^, in which the core idea is that there is a fundamental need to model the associations or statistical regularities in the world in order to optimise predictions and iteratively minimise prediction errors^7^. Broadly speaking, predictive processing accounts have framed hallucinations in terms of an over-weighting of top-down or recurrent connections, such that predictions generate perceptions despite a lack of sensory evidence^8–10^. Conversely, delusions have been suggested to arise initially from an over-weighting of bottom-up prediction error signals, which stimulate formation of new beliefs to account for the perceived inadequacy of existing ones^11–14^. More recently, a consideration of the precision or gain of both the prediction and the prediction error^15–17^ has extended these models. Overall, the hierarchical predictive processing approach appears to provide more comprehensive views on how perturbations in perception and learning may underlie psychotic illness^18–20^, and has illuminated potential neural mechanisms of psychotic symptoms^16^.

Signal precision is an important consideration in how an agent successfully models its world by striking a balance between flexibility and stability. While prediction errors provide the fundamental drive to updating, many environmental regularities are probabilistic, meaning that some prediction errors are inevitable and should not directly drive belief updating^21^. But, at the same time, favouring a learned model when there has been a genuine change in these regularities would be detrimental^6^. Achieving a balance is important to interacting optimally with our environment. This balance depends on estimated precision (or reliability) of the signal and may be disrupted in psychosis^22–24^. One useful approach to exploring this balance is through the use of reversal learning tasks in which associations are learned and then experimentally changed. Such tasks require participants to use an adaptive policy, maintaining particular responses in the face of probabilistic errors, while retaining a readiness to relearn this when the change occurs. A recent systematic review of a wide range of reversal tasks in people with psychosis suggests fundamental difficulties in adapting to volatile environments associated with a reduced sensitivity to experimentally-manipulated environmental volatility^25^.

In previous work^26^, we used a probabilistic learning task in order to explore how ketamine, a drug model of psychosis^27–31^ affected learning and updating of stimulus–response-outcome associations in healthy participants. Our task requires participants to maximise reward and minimise loss by learning appropriate responses to stimuli that were probabilistically related to monetary outcomes. In addition, there were occasional reversals of these cue-outcome contingencies. To perform the task successfully, participants needed to be able to ignore the probabilistic errors, maintaining their response strategy despite the fact that this would occasionally produce an undesirable outcome. However, because of the occasional contingency reversals, they also had to be able to change their strategy if it was no longer the optimal one. Ketamine was associated with difficulty in capitalizing on prevailing contingencies, i.e. with maintaining optimal policy in the face of probabilistic errors. Computational modelling of task performance suggested that this failure was underpinned by a shift in how a “confidence” parameter modulated responding. In essence, this means that ketamine attenuated the ability to persist with a response that had been successful in recent trials.

The key question is whether this alteration, which can be conveniently estimated in the context of a pharmacological model, would also be found in clinical groups with, or at risk of, psychosis. We therefore explored precisely the same task, using the same computational modelling procedure^26^, in relevant clinical populations representing several stages of psychotic experience. It has been suggested^30^ that ketamine provides a model for the early, emergent features of psychosis and we predicted that behavioural and computational changes in the at-risk mental state and first episode psychosis groups would most closely mimic those found under ketamine. Conversely, ketamine has also been noted to be unusual among drug models of schizophrenia in producing negative symptoms^27–29,31^ which might predict a resemblance to findings in our TRS group. We carried out two complementary patient studies. *Study 1* explored performance in patients whose subclinical psychotic-like experiences corresponded to an At-Risk Mental State (ARMS), patients with first episode psychosis (FEP), and matched healthy controls (HC1). *Study 2* analysed performance in participants with treatment-resistant, chronic schizophrenia (TRS) and matched healthy controls (HC2). In short, we wished to determine whether the previously-reported alterations in confidence-modulation of learning-rate and choice temperature produced by ketamine replicated across different stages and severities of psychosis.

## Results

### Behavioural results (study 1): ARMS, FEP and matched controls

#### Accuracy and risk tendencies

For every trial (see "Methods" section below), participants responded to a cue by deciding between a “risky” option (betting a larger sum (£1)) and a “safer” option (betting a smaller amount (10p)). Visual feedback reminded them of their choice, and then informed them of the outcome (‘win’ or ‘lose’ whatever they had chosen to wager). The expected value of choosing either of these options on a particular trial depended on the cue shown. At any given time during the experimental session, one cue was statistically associated with a high probability of a ‘win’ outcome (P(Win) = 0.8) and thus indicated that the riskier choice was optimal. The other cue was, meanwhile, statistically associated with high-probability loss outcomes (P(Win) = 0.2) and therefore signalled that it would be optimal to choose the “less risky” (10p) bet. Choices were considered ‘accurate’ if they conformed to what would, conditional upon the prevailing cue-outcome contingencies, yield greater expected return on the present trial. That is, the optimal choice conditional upon the current statistical relationship between the presented cue and the probable outcome—regardless of whether the trial happened to be one of the minority (20%) of trials that violated these contingencies—was considered ‘accurate’, since it conformed to the general strategy that would maximise gains and minimise losses throughout the current block of the task.

Groupwise averages of the overall response accuracy within HC1 (μ = 0.766; s.d. = 0.117); ARMS (μ = 0.693; sd. = 0.130); and FEP (μ = 0.648; s.d. = 0.118) differed significantly from one another [one-way ANOVA: df = 2,79; F = 6.66; *p* = 0.002]. Post hoc t-tests (Tukey–Kramer (TK) corrected) found patients with FEP were significantly less accurate than controls (*p* = 0.001), but were not significantly less accurate than those with ARMS (*p* = 0.341). The tendency for accuracy in the HC1 group to exceed that of the ARMS group was non-significant (*p* = 0.062).

The proportion of all responses that were ‘risky’ choices (i.e. trials on which participants wagered £1 rather than the alternative ‘less risky’ option of 10p), averaged within each group, was for HC1 43% (s.d. = 0.066), for ARMS 48% (s.d. = 0.086) and for FEP 42% (s.d. = 0.111). Proportion of risky choices was not significantly different between groups [Kruskal–Wallis (KW) ANOVA: df = 2,80; W = 5.808; *p* = 0.055].

### Choice switch tendencies

**Figure 1:**
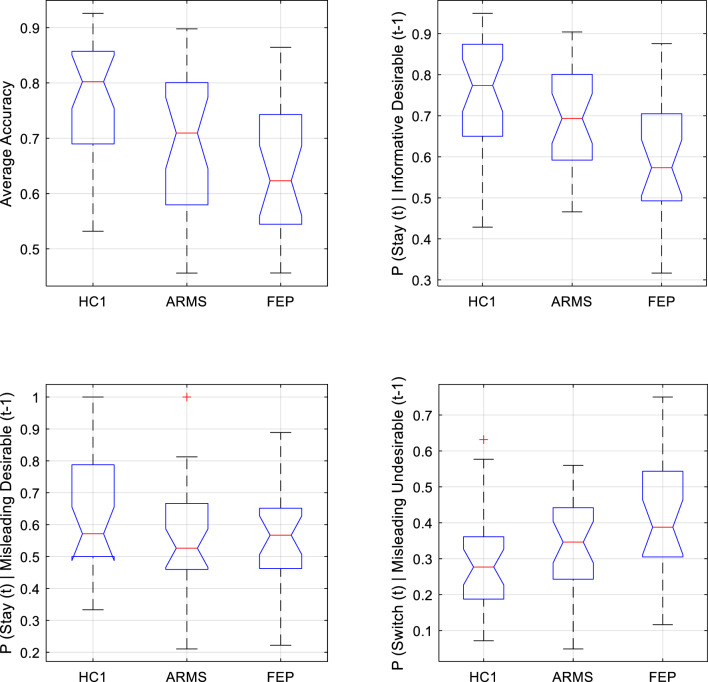
Boxplots showing median (), interquartile range (), and range () of key behavioural outcomes from *Study 1,* plotted separately for Healthy Control (HC1), At-Risk Mental State (ARMS) and First Episode Psychosis (FEP) groups. Any outlying data points within a group are plotted individually (). *Top left* Accuracy was significantly lower in FEP than HC1, and was lower at trend-level in ARMS than HC1. *Top right* Behavioural sensitivity to informative (high-probability) desirable feedback. The tendency to adaptively stay with an optimal strategy following desirable feedback was significantly lower in FEP than in both HC1 and ARMS groups (who did not significantly differ from one another in this regard). *Bottom left* Behavioural sensitivity to misleading (low-probability) desirable feedback did not differ between groups. *Bottom right* Behavioural sensitivity to misleading (low-probability) undesirable feedback. FEP participants were significantly more likely than HC1 participants to inappropriately ‘switch’ following low-probability undesirable feedback.

We operationally define outcomes according to whether they were more or less desirable than the counterfactual possibility that would have occurred, given the cue presented, had the alternative wagering option been chosen. More desirable outcomes entailed receiving the maximal gain or minimum loss on a given trial (i.e. receiving £1 rather than 10p, or losing 10p rather than £1). As discussed above, trials could also be categorised according to the optimality or correctness of the choice made. Crucially, all possible permutations of choice accuracy and outcome desirability were possible. Thus, a correct selection of the risky option could be followed by winning the (more desirable) £1 outcome or, on 20% of trials, by the less desirable outcome of losing a whole pound. If the participant erroneously responded to the relevant cue by choosing the non-risky response, they would usually receive a less desired outcome than the correct response would have garnered (i.e. would win only 10p rather than the £1 that would have been the outcome had they plumped for the alternative option in making their wager). On 20% of such trials, however, they would receive a more desirable outcome for their erroneous choice than the correct one would have yielded (i.e. if the low-probability ‘loss’ event happened to occur, then the counterfactual case in which they had made the correct choice relative to the prevailing cue-outcome contingencies, in this particular instance would have yielded an even larger monetary loss of £1). The same possibilities were afforded by the cue signifying optimality of a non-risky choice (in this case the more desired outcome was losing 10p while the less desired outcome was losing £1). A subject’s propensity to interpret (less) desirable monetary outcomes (losses of £1 or wins of 10p) as indicative of a genuine need to alter their current response strategy could be assayed, behaviourally, by their propensity to ‘switch’ to an alternative strategy on the trial immediately following such events. Similarly, the tendency for recent indications of performance success to promote policy stability (i.e. the tendency to ‘stick’ with a seemingly successful strategy) was assayed behaviourally by examining the tendency to stay with the same response strategy (rather than switching) on the next trial after receiving a relatively desirable outcome (losses of only 10p or wins of £1). We analysed switch behaviour (i.e. tendency to change one’s choice on the subsequent presentation of the same cue or to repeat the choice when the alternative cue was next presented) for each of the four permutations: (i) correct choice, more desirable outcome; (ii) correct choice, less desirable outcome; (iii) incorrect choice, more desirable outcome; (iii) incorrect choice, less desirable outcome. We describe group comparisons for each of these four permutations below:

(i) *Correct choice, more desirable outcome*: the optimal behaviour here is to stick with the same choice strategy on ensuing trials. As seen in Fig. 1 (top-right) This differed significantly across groups (one-way ANOVA; df = 2,80; F = 8.793; *p* < 0.0001): pairwise comparisons (TK-corrected) found it was lower in FEP than both HC1 (*p* = 0.0002) and ARMS (*p* = 0.0369), but did not differ between ARMS and controls (*p* = 0.1968). In short, FEP participants tended to shift from an optimal choice strategy even though it had received the more desirable outcome.
(ii) *Correct choice, less desirable outcome*: here, the optimal behaviour is to stick with the same choice despite the experience of a less desirable outcome. There was a significant group difference in probability of inappropriately shifting strategy. [One-Way ANOVA: df = 2,80; F = 4.96; *p* = 0.0093]. Post-hoc pairwise comparisons (TK-corrected) found FEP participants were more susceptible to such “surprise-shift errors” than HC1 participants (*p* = 0.0066), while the ARMS group did not differ from FEP (*p* = 0.137) nor HC1 (*p* = 0.423) in this regard (see Fig. 1, bottom-right). That is, FEP participants appeared less likely than controls to maintain an optimal strategy in the face of less desirable feedback.
(iii) *Incorrect choice, more desirable outcome*: there was no difference in tendency to shift strategy following choices that were incorrect, but (having being made on one of the minority (20%) of trials on which the prevailing probabilistic relationship between a cue and its typical outcome happened to be violated) had nevertheless received the more desirable outcome (one-way ANOVA, df = 2, 79; F = 2.44; *p* = 0.0939).
(iv) *Incorrect choice, less desirable outcome*: The groups did not differ in their probability of appropriately shifting away from (as opposed to persevering with) an incorrect strategy on trials immediately following an informative undesirable outcome (One-way ANOVA: F(2,80) = 0.4866, *p* = 0.617).

In brief, these analyses demonstrate a reduced tendency in FEP participants, compared to controls, to maintain the optimal choice strategy. While ARMS participants were numerically intermediate between HC1 and FEP, they did not significantly differ from either group. The findings are noteworthy in relation to the overall conclusions from the behavioural analysis of healthy participants under ketamine administration, which were that that ketamine reduced the ability to stabilize behaviour in the face of probabilistic (misleading) unexpected outcomes.

### Computational results (study 1): ARMS, FEP and matched controls

Variational Bayes analysis^32^ strongly supported the hypothesis that groups did not differ in model frequencies (P(y|H =) ~  = 1). We therefore pooled HC1, ARMS and FEP data for submission to random-effects Bayesian Model Comparison. The best-fitting model was a hierarchical model that implemented optimality-based confidence updating and used confidence to modulate learning-rate and choice temperature with separate weights. This model’s ‘exceedance probability’ (the probability it was more frequent than any other in the modelspace) was ep = 0.9998. Its “protected exceedance probability’’ (an extension of this notion, controlling for the possibility that one model may occur more frequently than all others simply by chance^32^) was pxp = 0.9995. Family-wise analysis found strong support (ep ~  = 1) for those models whose lower-level reinforcement learning algorithm was that of this winning model (i.e., symmetrical updating of cue-values according to outcome valence, reflecting an appreciation of the task structure) over less sophisticated alternatives (i.e. over families of models that updated only the seen cue’s value on each trial, and/or updated cue-values more from |£1| outcomes than equivalently informative |10p| outcomes). When families were defined instead by how confidence was updated, family-wise analysis found strong evidence in favour of optimality-based “surface-monitoring” (ep ~  = 1). Finally, comparing families defined by how confidence was used to modulate learning rate and/or choice temperature found strong support for the family of models in which confidence modulated both these lower-level parameters with separate weighting-factors (ep ~  = 1). These family-wise analyses support the validity of model 26, which uniquely occupies the intersection between the winning reinforcement learning, confidence-monitoring, and confidence-modulation families***.***

**Figure 2:**
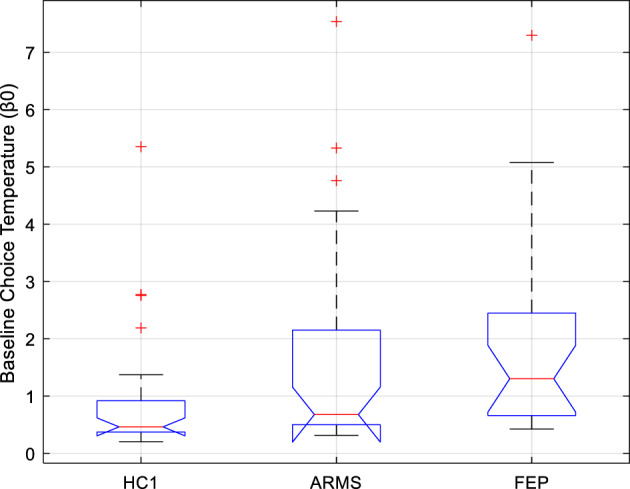
Boxplots showing the median (), interquartile range (), and range () of *Study 1* subjects’ baseline choice temperature free parameter (β_0_) under the winning computational model, plotted separately for Healthy Control (HC1), At-Risk Mental State (ARMS), and First Episode Psychosis (FEP) participants. Any outlying values of β_0_ within a group are plotted individually (). Compared to the control group (HC1), there was a significant elevation of β_0_ in patients with FEP, and a trend-level tendency towards elevated β_0_ in patients with ARMS.

Contrary to what we hypothesized based on previous ketamine findings^26^, neither of the two free parameters controlling confidence-modulation of lower-level parameters differed between groups. Of the winning model’s five free parameters, groups differed significantly only in baseline choice temperature β_0_ (Kruskal–Wallis ANOVA; df = 81,2; χ^2^ = 12.7; *p* = 0.0017). See Fig. 2. This remained significant following correction for multiple comparisons. Pairwise comparisons (TK-corrected) found median β_0_ was significantly higher in FEP than HC1 (*p* = 0.001), tended to be higher in ARMS than HC1 at trend-level (*p* = 0.067), and did not differ between ARMS and FEP (*p* = 0.339)***.***

Median β_0_ did not differ between patients who were taking antipsychotics, and patients who were not (*P* = 0.951; H = 0; z = 0.06, rank sum = 377). Further, among the former subgroup of patients, antipsychotic dose (in chlorpromazine equivalent units^33^) did not correlate with β_0_ (n = 16; ρ = 0.127; *p* = 0.640). Thus, the significant group difference does not seem to reflect a medication effect.

### Summary

The computational analyses suggested that, despite the behavioural resemblance between FEP and ketamine—in terms of a reduced tendency to stick to an optimal strategy when faced with probabilistic undesirable outcomes, there are computational differences: notably, in the current study, groups differed significantly only in one specific free parameter: baseline choice temperature β_0_ was lower in the FEP group (who also showed worse performance) than the HC1 group. In contrast to the previous study, no differences were observed across group in terms of the confidence weighting parameters.

The winning model here was largely identical to the one previously observed^26^, in that all three groups approached the task using a cognitive strategy involving: (1) maintaining a meta-level estimate of confidence in lower-level beliefs about values of cues; (2) updating this confidence estimate dynamically using outcome optimality as a teaching signal; and (3) deploying confidence to flexibly influence learning rate and choice temperature, using distinct weighting factors to separably control these (this latter finding represents the single point of departure between the winning model here and that of the previous study, which did not differentiate separate parameters for confidence-modulation of learning versus decision-making). An important caveat is that parameter recovery for four of the parameters was poor and only choice temperature, β0, was well-recovered from simulation data.

### Behavioural results (study 2): TRS and matched controls

#### Accuracy and risk tendencies

**Figure 3:**
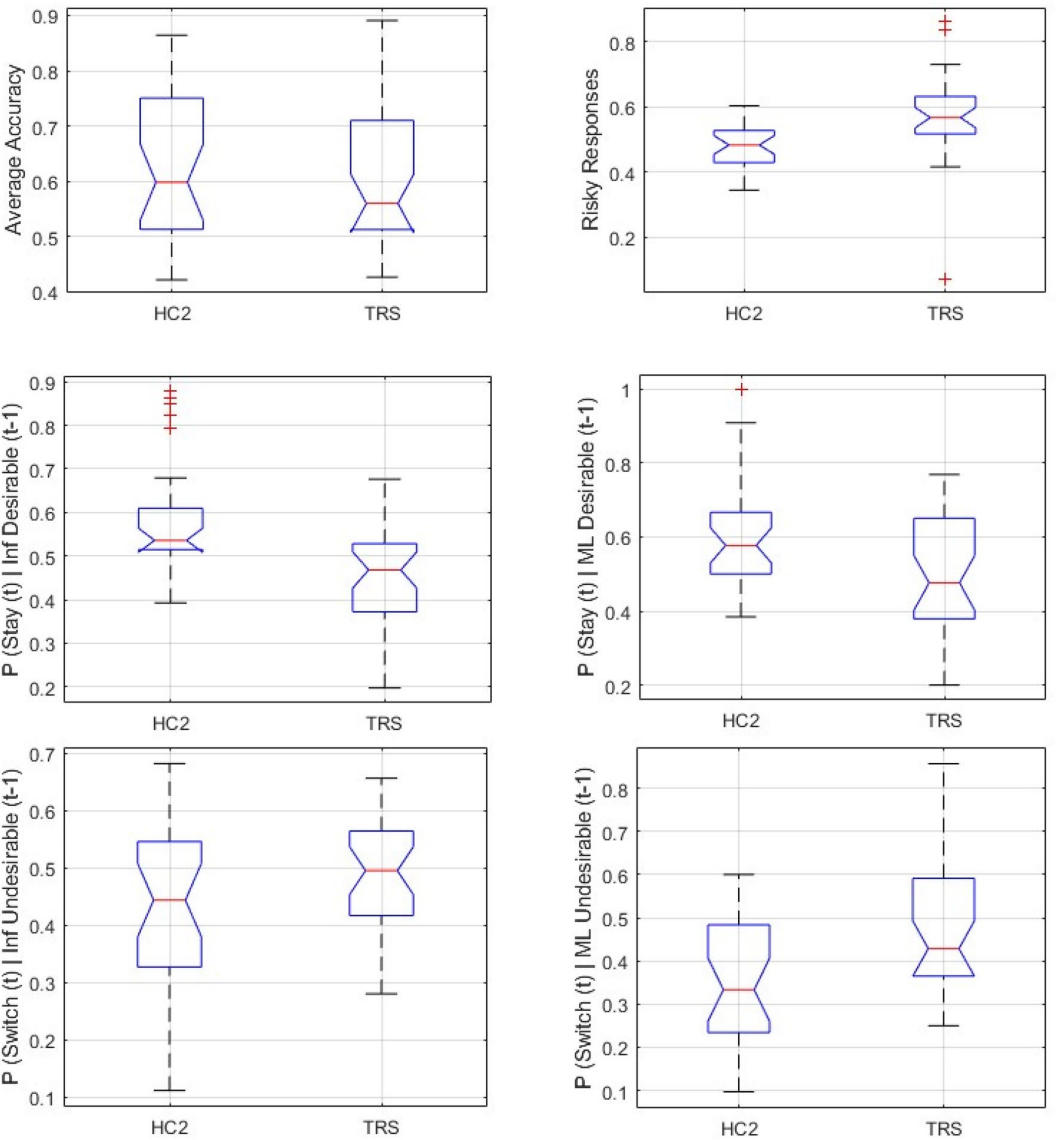
Boxplots showing median (), interquartile range (), and range () of key behavioural outcomes from *Study 2,* plotted separately for Healthy Control (HC2), and Treatment-Resistant Chronic Schizophrenia (TRS) groups. Any outlying data points within a group are plotted individually (). *Top left* Average accuracy did not differ significantly between HC2 and TRS. *Top right* Average proportion of all responses that were ‘Risky’ was significantly higher in TRS than HC2. *Middle Left* Compared to controls, TRS is associated with significantly reduced tendency to adaptively stay with an optimal strategy following high-probability (Informative, ‘Inf’) desirable feedback to a correct response. *Middle Right* Compared to controls, TRS is associated with significantly reduced propensity to inappropriately stay with a suboptimal strategy following low-probability (Misleading, ‘ML’) desirable feedback to an incorrect response. *Bottom Left* Compared to controls, TRS is associated with significantly elevated tendency to adaptively switch away from a suboptimal strategy following informative (‘Inf’) undesirable feedback (left) to an incorrect response. *Bottom Right* Compared to controls, TRS is associated with significantly elevated propensity to inappropriately switch away from an optimal strategy following Misleading (‘ML’) undesirable feedback to a correct response.

Accuracy (see Fig. 3 top-left) in the TRS group (μ = 0.603; s.d. = 0.123) was not significantly lower than accuracy in the HC2 group (μ = 0.628;s.d. = 0.133) [One-way ANOVA df = 1;58; F = 0.53; *p* = 0.469]. However, the proportion of risky choices (see Fig. 3 top-right) was significantly higher in the TRS (μ = 0.569; s.d. = 0.138) than in the HC2 group (μ = 0.479; s.d. = 0.07) [unpaired t-test, unequal variances, two-tailed: df = 52.617; t = 4.562; *p* < 0.0001].

#### Choice switch tendencies

As with Study 1, we examined tendencies to switch choices according to the four possible permutations of trial.

(i) *Correct choice, more desirable outcome*: as described above, the optimal behaviour here is to stick with the same strategy on the ensuing trial. However, TRS participants were more likely to inappropriately shift from a choice that had, on the previous trial, received informative desirable feedback—a tendency which differentiated them significantly from HC2 participants (one-way ANOVA: df = 1, 58; F = 14.907; *p* < 0.0003). See Fig. 3 middle left.
(ii) *Correct choice, less desirable outcome*: here, the optimal behaviour is to stick with the same choice despite the experience of a less desirable outcome. TRS was associated with significantly greater tendency to incorrectly switch away from the optimal strategy, i.e. to switch response when the preceding trial was associated with a correct response but an undesirable outcome (one-way ANOVA df = 1; 58; F = 5.6128; *p* = 0.0212). See Fig. 3 bottom right.
(iii) *Incorrect choice, more desirable outcome*. The TRS group were more likely than the HC2 group to shift from a choice that, on the preceding trial, had received a ‘misleadingly’ desirable outcome (one-way ANOVA: df = 1,58; F = 7.99; *p* = 0.0064). See Fig. 3 middle right. Note that, in this respect, they showed a more optimal tendency than the controls (although, in the context of their overall elevated tendency to shift choices, this may not signify a true improvement in performance, which was indeed comparable in terms of overall accuracy between TRS and HC2 groups).
(iv) *Incorrect choice, less desirable outcome*: The TRS group were more likely than controls to correctly switch after undesirable feedback following a suboptimal response (one-way ANOVA: df = 1,58; F = 7.76; *p* = 0.0072). |See Fig. 3 bottom left. Again, though optimal, this tendency should be considered in the context of their overall increased tendency to switch.

**Figure 4:**
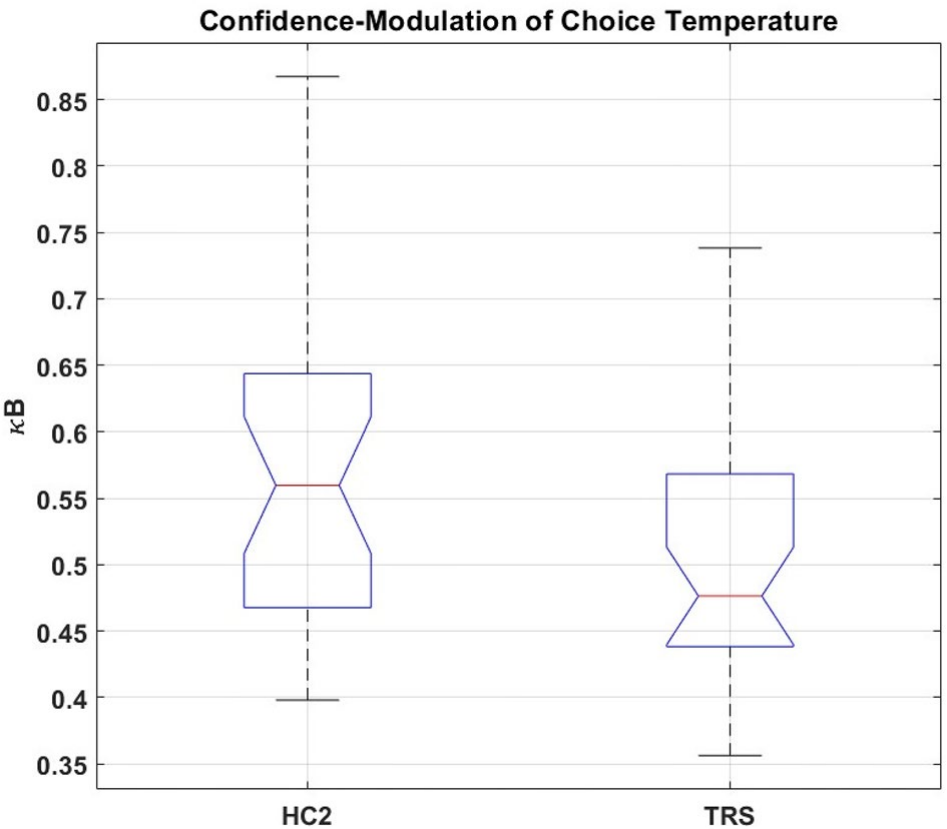
Boxplots showing the median (), interquartile range (), and range () of *Study 2* subjects’ values of κ*B* (the free parameter corresponding to the weighting factor for trialwise confidence-modulation of choice temperature, under the winning computational model) plotted separately for Treatment-Resistant Chronic Schizophrenia (TRS) and Healthy Control (HC2) groups. Any outlying values of κ*B* within a group are plotted individually (). Average κ*B* was significantly lower in TRS than HC2.

In brief, for all permutations of choice and outcome, TRS participants showed an increased tendency to change their strategy across successive trials. While in some cases, this was optimal, and may indeed have contributed to their preserved accuracy relative to matched controls, the tendency proved disadvantageous in that it was to the detriment of maintaining optimal responding across the study session and, as with the previous ketamine study and the ARMS participants in study 1, suggest that the clinical group showed a reduced ability to stabilise responding in a probabilistic setting (Fig. 4).

### Computational results (study 2): TRS and matched controls

Variational Bayes analysis^32^ strongly supported the hypothesis that model frequencies did not differ between TRS and HC2 groups (P(y|H =) = 0.9998). Pooling both groups’ data and submitting it to random-effects Bayesian Model Comparison to estimate the most likely distribution of models in this sample, we replicated the findings of *Study 1*. Once again, the data were best fit by the hierarchical model using outcome desirability as a teaching signal for confidence, which modulated both learning rate and choice temperature with different weights. The ‘exceedance probability’ of this winning model within the present sample was ep = 0.9103. Protected exceedance probability (pxp) for the winning model was pxp = 0.6797. Thus, the probability that in this sample, this best-fitting model was more frequent than any other “not by chance”^32^ perhaps seems dubiously low. However, the previous pharmacological work^26^ and *Study 1* both found strong evidence for a significant difference in these models’ frequencies. Further, the most frequent model in *Study 1* was the same model as best fit *Study 2’s* independent dataset here, and furthermore was identical, in all respects save for not differentiating between parameters κ*A* and κ*B*, to the winning model in the ketamine study^26^ (possibly due to power limitations stemming from that previous study’s smaller sample size). Thus, the more a priori probable assumption that (consistent with previous findings) “these models differ from one another in frequency” renders ep a more appropriate metric (and pxp overly conservative) in this case.

Family-wise analyses replicated the findings from the identical family-wise analyses reported for *Study 1*’s separate dataset: concurring with the random-effects model comparison in supporting the model’s validity within the HC2/TRS sample. There was strong support for the family whose reinforcement learning algorithm used outcome valence to update both cues symmetrically on each trial (ep = 0.9735); for the family updating confidence according to outcome desirability as per Eq. (4) (ep ~  = 1); and for the family using confidence to modulate learning-rate and choice-temperature with separate weights (ep = 0.9852).

Of the winning model’s five free parameters, four showed no significant group difference. The exception was κ*B*, the weight with which confidence modulates choice temperature: average κ*B* was higher in HC2 than TRS (one-way ANOVA, df = 1,58; F = 4.235; *p* 0.0437).

Patients’ clozapine dose did not correlate with κ*B* (ρ = 0.0160; *p* = 0.929), nor did their current clozapine level (ρ = 0.123; *p* = 0.496). Whether effects of other antipsychotic medication, in those patients who were additionally taking them, might confound our finding’s interpretation was investigated using an unpaired t-test (unequal variances). The subgroup of patients taking other (typical) antipsychotics as well as clozapine did not differ, in average κ*B*, from those patients taking only clozapine (t = − 0.302; df = 31.8; *p* = 0.765). Thus, the difference between HC2 and TRS in κ*B* does not seem attributable to antipsychotic medication.

### Summary

In contrast to ARMS and FEP, people with TRS showed a reduction in average κ*B*: the free parameter governing the weight with which meta-level confidence influences choice temperature. Thus, TRS resembled the pattern of reduced confidence-modulation observed under ketamine^26^. These interesting observations must be tempered by two caveats: first, simulation-based parameter recovery for κ*B* was poor and, second, the groupwise difference did not survive a correction for the five parameter comparisons. For these reasons, though we find this apparent overlap between computational alterations across TRS and ketamine interesting, we treat it cautiously.

### Exploratory analyses of relationship between task performance and delusional symptoms

These exploratory analyses focused on the question of whether the computational parameters showing significant group differences (i.e. *β0* in FEP and *κB* in TRS) showed any relationship to clinical features.

### FEP and ARMS: no relationship between delusions and β0

There was no significant correlation between delusional symptom severity (PANSS P1^34^) and baseline choice temperature β_0_ in the FEP group ($\sigma$ = − 0.227, *p* = 0.398), nor in the ARMS group ($\sigma$ = 0.092; *p* = 0.669). There was no correlation between PANSS P1 delusion severity and κ*B* in participants with FEP ($\sigma$ = 0.209; *p* = 0.391), nor ARMS ($\sigma$ = 0.055; *p* = 0.799). Delusional ideation measured by the CAARMS (non-bizarre ideas + unusual thought content)^35^ showed no significant relationship with β_0_ in ARMS ($\sigma$ =− 0.178; *p* = 0.452), nor in FEP ($\sigma$ = 0.176, *p* = 0.486), nor in both these patient groups together ($\sigma$ = 0.138, *p* = 0.410)).

### Higher κB in TRS associated with presence of delusions

Due to the bimodal distribution of delusional symptoms within the TRS sample, patients were divided into two sub-groups based on delusional symptom severity. Average *κB* was significantly lower in patients with non-delusional (PANSS P1 = 1) TRS than with delusional TRS (PANSS P1 = 3–4) (one-way ANOVA; df = 1,18; F = 5.8; *p* = 0.027).

## Discussion

Across two studies we examined probabilistic reversal learning in three clinical groups. *Study 1* characterised learning in at-risk mental state (ARMS) and first-episode psychosis (FEP). *Study 2* examined people with what has been termed treatment-resistant schizophrenia (TRS), i.e. participants diagnosed with schizophrenia and treated with clozapine due to a lack of efficacy of standard antipsychotic treatments. We used behavioural and computational analyses to characterise alterations in learning and choice. We are cautious about the computational analyses for reasons discussed below, and will begin by discussing the behavioural findings across the two studies.

The behavioural results suggest a distinction between ARMS, FEP and TRS phases of psychotic experience, in terms of how patients within these groups approach learning and decision-making under uncertainty. While FEP was associated with a significantly reduced overall accuracy level, there was no significant group difference in overall propensity to make risky choices, despite a numerical tendency towards riskier behaviour in the ARMS group—whose accuracy level was intermediate between FEP and controls, and was different at a trend-level from the control group. Conversely, TRS participants did not differ on overall accuracy from their matched control group, but did show a significantly greater tendency to make riskier choices.

A more detailed analysis of trial-to-trial behaviour considered tendencies to shift from one behavioural strategy for cue-guided choice to another, in response to probabilistic feedback. Here, we observed that FEP participants were more prone than controls to switch away from optimal responding, after experiencing both more desirable and less desirable outcomes. They did not show an increased tendency to switch away from a sub-optimal pattern of choice, irrespective of the desirability of the outcome on the previous trial. This may be at the root of their reduced accuracy, and accords with the behavioural impact observed in healthy participants undergoing acute ketamine challenge. Conversely, patients with TRS showed increased behavioural “switch” tendencies irrespective of the optimality of their previous choice or the desirability of its outcome, suggesting reduced stability of responding which is also a characteristic of chronic schizophrenia. Interestingly, TRS participants showed no concomitant reduction in overall accuracy compared to controls.

Overall, the behavioural findings in the clinical groups echo the previous observation that ketamine was associated with a reduced ability to stabilise responding within a probabilistic environment in which even optimal responses will occasionally be followed by less desirable outcomes. The apparently paradoxical observation that the TRS participants, whose choice behaviour was markedly more changeable (or less stable) than matched controls’, nevertheless showed preserved accuracy may be accounted for by these patients’ general tendency to switch towards as well as away from optimal responses, which contrasts with the FEP group’s more specifically elevated sensitivity to probabilistic undesirable feedback following optimal choices. It is possible too that the generally increased flexibility (or reduced stability) of the TRS group’s responses had a mitigating effect on the disruptive effect of probabilistic reversals which occurred on three occasions over the study session. That is, relatively unstable responding can confer a brief advantage when the environment is volatile.

The ensuing computational analyses sought to determine the deeper processes underpinning more superficial behavioural observations, and focused on whether the TRS and FEP groups’ behavioural resemblance to healthy participants under acute ketamine administration on this task was accompanied by comparable alterations in the computational parameters associated with ketamine. In brief, the prior work^26^ suggested that ketamine infusion reduced the capacity to stay with an optimal behavioural policy when confronted with probabilistic undesirable outcomes, and computational modelling indicated a reduction in the degree to which meta-level confidence modulated lower-level reinforcement learning parameters so as to promote stricter adherence to policies that were, based on recent performance, more likely to be optimal.

As in the ketamine study, the computational model most successfully capturing choice data for studies 1 and 2 suggested that all groups approached the task by maintaining an estimate of meta-level confidence in their lower-level beliefs, and updated confidence dynamically using outcome (un)desirability as a teaching signal, with confidence growing in proportion to the number of recent desirable response-feedbacks. However, it should be noted that this winning model included multiple free parameters (in order to fully recapitulate the previous study’s analysis) which meant that parameter recovery was overall poor. Our discussion below is therefore tempered with caution.

Our prediction was that the ARMS and FEP groups would show computational alterations resembling healthy participants treated with acute ketamine. However, this was not the case: while ketamine was primarily associated with reduced influence of current confidence over learning and behavioural policy, *Study 1* showed that confidence-weighting parameters were not altered in ARMS or FEP compared to controls. Instead, the free parameter which in this hierarchical winning model corresponds to ‘baseline’ choice temperature, β_0_, was significantly elevated in FEP (and numerically, but not significantly, in ARMS) compared to controls. Choice temperature may signify relative randomness of responding^36^, and the increase in this parameter among FEP participants accords with our behavioural analysis demonstrating a relative failure to consistently maintain an optimal response pattern, an effect that was even more pronounced in TRS, the latter according with the conclusions of a recent systematic analysis^25^. Second**,** despite their more advanced illness stage, the TRS group (unlike ARMS and FEP) showed a computational perturbation resembling that produced by ketamine: namely, a reduction in the degree to which current confidence level (as estimated by recent performance success)modulated choice temperature. (It should be noted that the ketamine model comparison findings differed in one respect from the present two studies’, in that they did not differentiate two separate weighting factors for confidence modulation of learning-rate and choice temperature^26^).

However, we emphasise here that, as mentioned above, while simulation-based analysis showed that recovery of the β_0_ parameter (altered in FEP) was good, it was poor for the parameter capturing the altered confidence-modulation in TRS (κ_B_), a failure that appears common to many papers in the field as parameter recovery is often not reported on^25^. This is an important area for improvement in future studies as successful parameter recovery is essential for the development of valid and interpretable models^37^. Furthermore, the difference in this confidence-modulation parameter between TRS and matched controls did not survive correction for multiple comparisons. Thus, while it is striking that the pattern in FEP and ARMS groups differed from that under ketamine, the apparent computational resemblance between ketamine and TRS should be treated cautiously.

Our experimental approach across these studies follows a growing interest in seeking to understand delusional beliefs in relation to observations from associative learning research. It is challenging to relate complex symptoms like delusions to underlying cognitive processes and, in this regard, insights from associative learning have proven attractive and useful by offering simple models of how an agent samples evidence from its environment and uses this to derive inferences about the associative regularities structuring that environment. This provides a powerful framework for developing theories of delusion formation. Early work by Miller, inspired by associative learning, considered psychosis in terms of a lowering of the threshold of evidence for updating beliefs based on new observations, leading to a “hyperactivity of associations”^38^. Learning theory was also central to powerful cognitive models of schizophrenia developed by Hemsley, Gray and others^39^ and subsequent neuroimaging work built on this to establish the presence of underlying perturbations in prediction error as a possible explanation for aberrations in belief updating in the context of associative learning tasks^11,12,40^. The link to prediction and prediction error, and to their underlying neurobiology, has been demonstrated across a range of tasks and techniques^40–44^. This body of work shares the perspective that it is instructive to conceptualise delusions in terms of the integration between predictions and sensory evidence^4,5,14,45,46^, and this has latterly included a consideration of how precision-based weighting of signals may provide a more complete framework for thinking about when and how new evidence (or prediction error) is used to update existing beliefs.

While a more comprehensive discussion of learning tasks in psychosis is beyond the scope of this paper, a recent systematic review of this literature suggested that, in reversal tasks such as ours, psychosis is associated with difficulties in reacting to changing contingencies: a phenomenon perhaps underpinned by relative insensitivity to environmental volatility, and by enhanced responses to irrelevant information^25^. Our findings are consistent with this—though they indicate that this characteristic pattern of disruption, in the context of different stages or forms of psychosis, may emerge from quite different underlying perturbations. Behaviourally, FEP and TRS are both associated with difficulties in sticking to an optimal strategy when it is challenged by unexpected and undesirable outcomes. Importantly, in TRS this general lability was reflected in elevated adaptive, as well as maladaptive, policy-shifting behaviour compared to controls. The disparities between the patterns of computational parameters, in the pharmacological model of early psychosis^26,47^ compared to Study 1’s ARMS and FEP patients, may reflect experimental differences: the task was administered twice in the previous study (once under ketamine and once under placebo), and although order was counter-balanced, findings might have been affected by between-session differences in participants’ overall familiarity with the task. Another explanation for the discrepancy between the clinical and pharmacological model computational findings is that ketamine was administered as a planned, acute, transient experience whereas the experiences of participants with ARMS and FEP have developed gradually, with corresponding adaptations in how these individuals update their beliefs over the course of weeks and months. While this differing temporal profile of experiences might account for the differences between ketamine and FEP, such an explanation raises the question of why people with TRS (whose experience of psychotic illness has been even more persistent and prolonged) show computational alterations that do resemble those observed under acute ketamine: that is, TRS was likewise associated with reduced confidence modulation of choice temperature. One speculative explanation is that progression from an acute (FEP) to a more chronic (TRS) state involves gradual adaptation to “persistent doubt”^26^. An initial period (in FEP) of more random choice and behaviour^48,49^ could lead to increased doubt about one’s ability to accurately predict the world. This would render confidence a poorer assay of how reliably true one’s current beliefs are likely to be. That is, as psychosis becomes more established, perhaps a shift could occur from doubting (and therefore updating) one’s current model of the world^26,50^, to doubting whether one can actually model the world successfully^51–53^. That is, confidence in one’s beliefs may no longer predict that choices based on these beliefs will reliably yield expected outcomes^54^. This, in turn, could manifest as partial uncoupling of confidence from choice temperature, as we observed in TRS. To put things simply, if FEP is characterised by a search for new priors to better predict the world, perhaps TRS is characterised by a sense that updating priors adds little to the world’s predictability.

From another perspective, our findings are also consistent with the idea that TRS represents a distinct subtype of schizophrenia^55^. Schizophrenia is defined as ‘treatment-resistant’ in cases where symptoms are not responsive to treatment with two or more antipsychotic (dopamine D2-receptor antagonist) medications. Thus, in contrast to treatment-responsive forms of psychosis, it may be secondary to a non-dopaminergic pathology, mediated more by glutamatergic dysfunction. In TRS, NMDA-R hypofunction is believed to play a greater ongoing role in symptom maintenance, via its consequent hyperglutamatergic cortical state^56,57^.

Overall, we replicated a previous study design and analysis to explore whether distinct stages of clinical psychosis might resemble the effects of an acute ketamine challenge on learning and decision-making under uncertainty. From a phenomenological perspective, ketamine’s effects appear more redolent of prodromal and early psychosis, and we did indeed observe that FEP was associated with a relative failure to stabilise choice behaviour in the face of probabilistic challenges. This instability of choice was more widespread in TRS and it was this more chronically unwell group who showed a greater resemblance to ketamine effects in the computational analysis. While, as discussed, we are cautious in interpreting the computational findings, our study shows the importance and value of thinking about psychosis in terms of its evolution over time since there are clearly both behavioural and computational distinctions between early and later stages of the condition.

## Methods

### Ethics declaration

The authors assert that all procedures contributing to this work comply with the ethical standards of the relevant national and institutional committees on human experimentation and with the Helsinki Declaration of 1975, as revised in 2008. Full informed consent from all participants was obtained in writing.

All experiments were designed and conducted in full accordance with local guidelines and regulations governing psychiatric research studies including human participants. *Study 1* was part of the Neuroscience in Psychiatry Network (NSPN) Neuroscience Clinical Adolescent and Adult Psychiatry Study (NCAAPS), under the approval of West of Scotland Research Ethics Committee 3 (NSPN) and Cambridgeshire 3 National Health Service Research Ethics Committee. Joint institutional sponsorship was provided by Cambridgeshire and Peterborough NHS Foundation Trust (CPFT) and University of Cambridge. *Study 2* was conducted under the approval of Cambridgeshire 3 National Health Service Research Ethics Committee, and jointly sponsored by CPFT and University of Cambridge.

### Study 1 sample (ARMS, FEP, HC1)

Data was obtained from n = 83 participants in the NCAAPS-Psychosis sample, which has been described in detail elsewhere^44^. Of these, n = 24 (20 M, 4F) had a diagnosis of first-episode psychosis (FEP); n = 29 (21 M, 8F) had an at-risk mental state (ARMS); and n = 30 were matched healthy controls (HC1) with no history of mental illness (16 M, 14F).

### Study 2 sample (TRS, HC2)

Data was obtained from n = 60 participants, of whom n = 31 were patients with chronic treatment-resistant schizophrenia (TRS) recruited from the NHS Clozapine Clinic in Cambridge. Inclusion criteria for the TRS group were (1) age 18–65 years, (2) no major physical or substance use comorbidities, (3) no changes to clozapine treatment within at least three months. Matched healthy control (HC2 group) participants were recruited from online bulletins, and through fliers posted in public spaces (e.g. community noticeboards and local businesses). HC2 group inclusion criteria were: (1) age and demographic match to existing TRS patient sample, (2) no history of psychiatric illness, (3) no major physical conditions and/or psychotropic medication use.

Of the 40 patients recruited for Study 2, useable datasets were obtained from 31 (six patient datasets were lost due to technical issues, and three were excluded during quality control due to evident failure to understand or implement the task instructions). One healthy participant was unable to complete the task due to a technical issue during testing, and a replacement HC2 participant was subsequently recruited as a suitably matched control for the relevant patient dataset.

All participants (across both studies) were able to speak and understand written English, gave full written informed consent, and were reimbursed for their time and effort (Tables 1, 2, 3).

### Study 2: treatment-resistance schizophrenia (TRS) and matched healthy controls (HC2)

**Table 1:** Mean (and standard deviation, s.d.) ages for participants in TRS and HC2 groups, and for all *Study 2* participants (male and female) within each of those two group, are indicated.

	Female	Male	Male and Female
TRS	N = 4 57.00 (s.d. 6.683)	N = 27 45.926 (s.d. 9.742)	N = 31 47.35 (s.d. 10.05)
HC2	N = 4 59.750 (s.d. = 5.188)	N = 25 51.700 (sd = 11.098)	N = 29 53.88 (s.d. 10.70)

**Table 2:** Mean and standard deviation (s.d.) clozapine dose in TRS group.

	Clozapine Dose Mean (s.d.)	Additional Typical Antipsychotics (AP)	Mean (sd) Typical AP (aripiprazole dose-equivalent)*
Female TRS (n = 4)	293.75 (82.60)	None (n = 2)	12.50 (10.61)
		Aripiprazole (n = 2)	
Male TRS (n = 27)	325.00 (127.48)	None (n = 13)	16.00 (7.12)
		Aripiprazole (n = 12) Amisulpride (n = 1) Sulpiride (n = 1)	
All TRS Patients (n = 31)	316.67 (110.81)	Clozapine Only: N = 15	15.17 (6.37)
		Any Typical AP: N = 16	

Indicated are numbers of patients with TRS taking additional typical antipsychotics (AP), the typical AP types taken, and typical AP daily dose-equivalent to oral ariprazole (mg) (the most frequent typical AP in this TRS sample).

**Table 3:** Mean and standard deviation (s.d.) for illness duration (years from onset of first psychotic episode), cognitive function assayed by the Brief Assessment of Cognition in Schizophrenia (BACS)^58^, and overall severity of symptom dimensions assayed by the PANSS structured clinical interview^34,59^.

	Female	Male	All TRS Patients
Illness duration (years)	28.00 (8.60)	24.28 (8.56)	24.65 (8.53)
BACS z-score^59^	− 1.26 (2.14)	− 1.37 (1.42)	− 1.36 (1.46)
PANSS Negative Symptoms Total	9.50 (3.54)	17.91 (7.80)	12.15 (10.31)
PANSS Positive Symptoms Total	12.00 (7.07)	12.05 (4.38)	12.04 (4.44)
PANSS General Symptoms Total	12.00 (13.86)	19.13 (13.68)	26.39 (6.52)
PANSS Disorganization Factor**	9.50 (2.12)	15.64 (4.55)	15.13 (4.70)

*Lower BACS z-score corresponds to more severe cognitive impairment in TRS.

#### Task description

Each trial began with a fixation cross (~ 500 ms), after which one of two cues (‘A’ or ‘B’) was presented for ~ 1750 ms. A question mark then appeared, prompting the participant to respond (within a window of opportunity ~ 1500 ms), after which a printed reminder of their choice (“Risky” or “Less Risky”) was displayed for ~ 750 ms. Finally, the outcome (monetary amount won or lost) was displayed for ~ 1750 ms. Cues A and B, for each participant, were two randomly selected elements from a set of 24 different Agamothodeian font characters. On each of 240 trials, one of these cues was randomly selected for presentation, and participants reported their choice between “risky” and “less risky” options by making one of two alternative motor responses corresponding to “risky” (£1) and “less risky” (10p) gambles on the trial’s outcome.

In *Study 1*, the relationship between “button press” (left/right) and ”choice” (risky/less risky) was randomised across participants within each group. In *Study 2*, the task was delivered on a Dell 15.6″ laptop (rather than in the scanner) and participants reported choices using key-presses (‘b’/ ‘z’) whose corresponding choice options were likewise randomized within HC2 and TRS groups.

Participants’ choices controlled only the variance of the outcome (i.e. whether it would involve a magnitude of 10p or £1), and had no influence over its valence (i.e. whether it likely to be a ‘win’ or a ‘loss’). Cues signified the probability of outcomes: one cue predicted an 80% chance of winning, while the other cue predicted an 80% chance of losing. Thus, one cue indicated that the optimal choice was the “Risky” option (betting £1) while the other cue indicated the “Less Risky” option (betting only 10p) was optimal.

A given cue-outcome contingency (e.g. P(loss|A) = P(win|B) = 0.8) was consistent for 60 trials before reversing.

In short, this design examined participants’ ability to learn what each cue indicated was the optimal response, to persist with this strategy despite occasional “misleading” undesirable outcomes, and to flexibly alter that policy whenever contingencies reversed.

### Behavioural analyses

**Figure 5:**
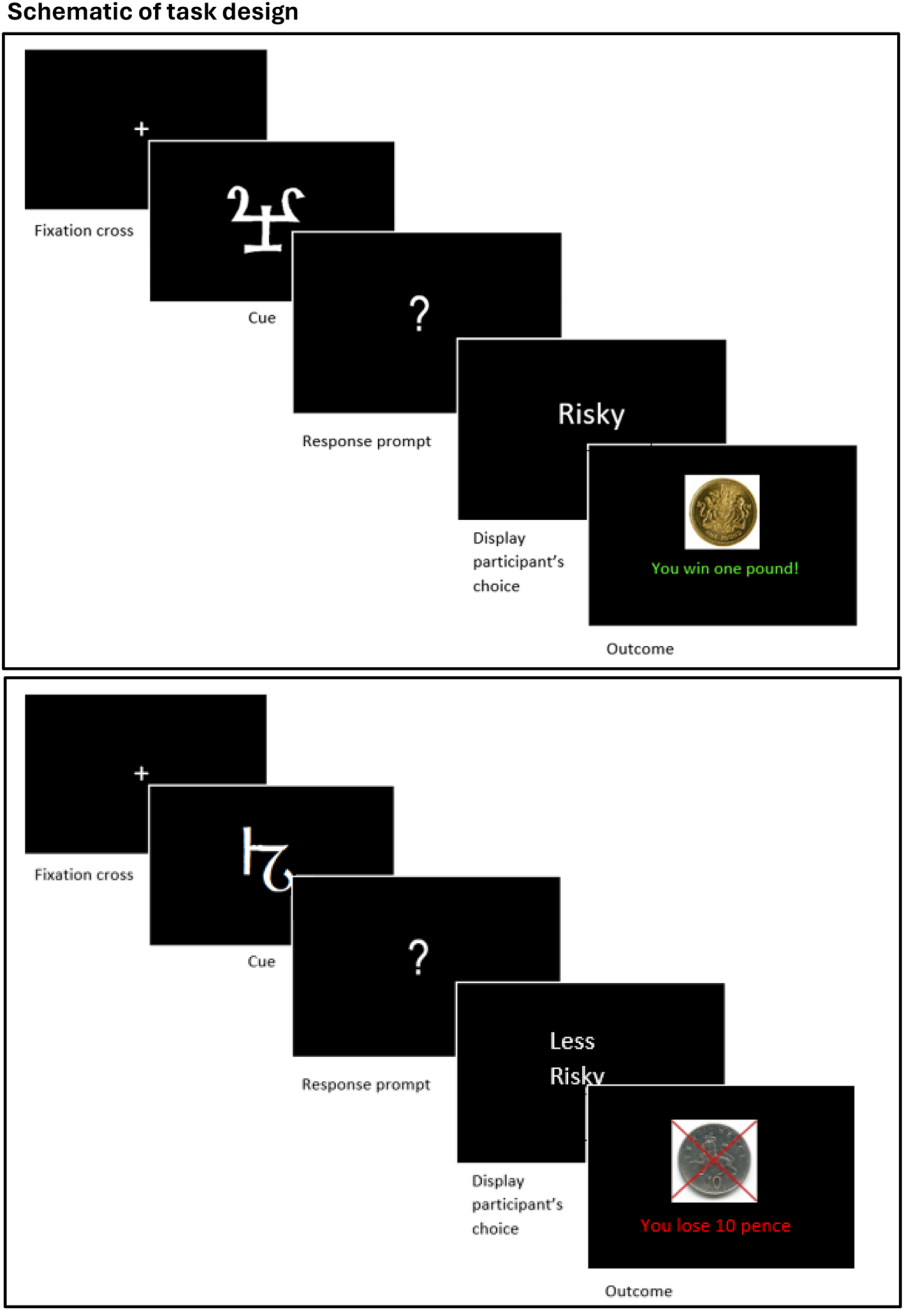
Upper Panel: Schematic illustration of a single trial (“good” cue). At cue onset, one of two abstract symbols is presented (P(cue A) = 0.5; P(cue B) = 0.5). Supposing the cue illustrated is currently the ‘good’ cue, then the ‘risky’ choice (to bet £1) is optimal, and the illustrated outcome is the most likely result of that choice (P(‘win’|’good’ cue) = 0.8). Lower Panel: Schematic illustration of a single trial (“bad” cue). At cue onset, one of two abstract symbols is presented (P(cue A) = 0.5; P(cue B) = 0.5). Supposing the cue illustrated is currently the ‘bad’ cue, then the ‘less risky’ choice (to bet only ten in-game pence) is optimal, and the illustrated outcome is the most likely result of that choice (P(‘loss’|’bad’ cue) = 0.8).

Overall performance was assayed, for each participant, as the proportion of all responses that were “accurate”, relative to the currently-prevailing reinforcement schedule (i.e. “less risky”|cue(P(win) = 0.2), and “risky” | cue(P(win =  = 0.8). See Fig. 5.

In behavioural analyses, the undesirability of an outcome (relative to the counterfactual outcome that would have occurred had the participant chosen the alternative wagering option) was used as a behavioural proxy of “surprise” associated with it. Assuming that choosing the risky (less risky) gamble corresponds to the subject’s expectation of reward (loss), “lose £1” and “win 10p” outcomes are both construable as surprising events that potentially can stimulate a ‘shift’ in behavioural policy detectable in the immediately following response.

Such a behavioural ‘shift’ was considered to have occurred if the same cue was seen, but a different choice made, on two consecutively played trials—or choice(t) ~  = choice(t + 1) while cue(t) =  = cue(t + 1)—or (alternatively) if having seen a different cue, the same choice was made, across two consecutive trials.

Before any parametric statistical testing, data was visually inspected, and submitted to a KS-test to confirm the normality assumption was not violated. Equality-of-variance between groups was assayed visually, and submitted to an F-test, to determine whether there was any violation of the equality-of-variance assumption. Wherever normality and equality-of-variance could be confidently assumed, one-way ANOVA was used.

In the instance where data in *Study 1*’s three groups was significantly different from normally distributed, a non-parametric variant (Kruskall-Wallis (KW) ANOVA) was used, as indicated in the text. In the instance where variances of HC2 and TRS groups’ data in *Study 2* was significantly different from equal, unpaired t-test under the unequal-variance assumption was used to compare them, as indicated in the text.

All statistical tests (across both studies) were two-tailed, with significance threshold *p* < 0.05*.

### Computational modelling

The computational analysis recapitulated that used to analyse data from this task in the previous within-subject study of healthy participants under placebo vs ketamine^26^. We fit 27 qualitatively different computational models to participants’ trialwise choice data. In three models, reinforcement learning occurred at a single level (at which beliefs about cue-values were stored, updated, and used to inform behavioural output) without any confidence-monitoring. The other 24 models were “hierarchical”, i.e. they additionally incorporated an estimate of meta-level confidence about these lower-level beliefs, using this confidence to modulate the lower-level parameters α_m_ (trialwise learning-rate for updating cue-value estimates) and/or β_m_ (trialwise choice temperature). This confidence-modulation rendered information more influential over belief-updating and/or response selection when it was likely to be reliable, compared to when it was more dubious.

Confidence was itself updated trialwise, with confidence-update rate γ (a free parameter 0 ≤ γ ≤ 1). In one family of hierarchical models, confidence was updated according to outcome “surprisal”: the lower-level prediction error’s absolute value (|δ(*t*)|). In another family of hierarchical models, confidence was instead updated according to outcome desirability (D(Outcome (t))). “Surprisal-based” confidence-updating decouples inferences about the validity of lower-level beliefs from the predictable consequences of one’s own action. Outcome-Desirability-based “surface-monitoring” may be favoured as a computationally-expedient heuristic alternative that makes use of salient information manifest in the outcome itself (its desirability/undesirability relative to the counterfactual amount that would have been won or lost, had the participant chosen the alternative wager) as a readily-available teaching signal: one that constitutes a proxy of beliefs’ validity by virtue of their causal relationship with behaviour (i.e. assuming one acts optimally conditional upon one’s current expectations, one’s actions are a reliable indicator of one’s beliefs: undesirable outcomes can thus usefully inform on the uncertainty of beliefs, to the extent beliefs determine actions predictably).

### Lower-level reinforcment learning algorithm model families (Eqs. 1–3)

Equation (1): ‘Basic’ Reinforcement Learning of Cue-Values

1 $$\begin{aligned} & V \left( { SeenCue\left( t \right)} \right)_{t + 1} = V\left( { SeenCue\left( t \right) } \right)_{t} + \alpha_{m} \left( { \delta \left( t \right) } \right) \\ & \delta (t) \, = \left( {MonetaryOutcome\left( t \right) {-} V\left( {SeenCue\left( t \right)} \right)_{t} } \right) \\ \end{aligned}$$

V(SeenCue(t)) = Value of the cue presented on trial t

$\alpha_{m}$ = learning rate on trial t

δ(t)﻿ = Prediction Error on trial t

The ‘Basic’ Reinforcment Learning (RL) algorithm for lower-level belief updating implemented a standard Rescorla-Wagner rule for trialwise prediction-error driven updating of only the seen cue’s estimated value (i.e., despite the symmetrical relationship between the two cues with respect to outcome probabilities, here belief updating occurred only for the cue that had been presented on the trial in question (SeenCue(t)) and prediction error, δ(*t*), was calculated as the difference between the actual monetary outcome received on that trial (i.e. MonetaryOutcome(t) could take values − 0.1, 0.1, − 1, or 1) meaning that larger-magnitude outcomes drove more learning than smaller ones (despite there being no dependency between the cue presented and the magnitude of its associated outcome, such that equivalent information was available from outcomes involving 10p as for outcomes involving £1).

Equation (2): ‘Outcome-Normalized’ Reinforcement Learning of Cue-Values

2 $$\begin{aligned} & V \left( { SeenCue\left( t \right)} \right)_{t + 1} = V\left( { SeenCue\left( t \right) } \right)_{t} + \alpha_{m} \left( {{ }\updelta \left( {\text{t}} \right){ }} \right) \\ & \delta (t) = \left( {OutcomeValence\left( t \right) {-} V\left( {SeenCue\left( t \right)} \right)_{t} } \right) \\ \end{aligned}$$

V(SeenCue(t)) = Value of the cue presented on trial t

$\alpha_{m}$ = learning rate on trial t

δ(t)﻿ = Prediction Error on trial t

In Eq. (2), the update-rule is identical to that of Eq. (1), but the calculation of ${\updelta }\left( {\text{t}} \right)$ (the prediction error term in that update-rule) relies on subtraction of the seen cue’s current valuation at the beginning of trial t from the valence (rather than the monetary value) of that trial’s outcome, reflecting the irrelevance of outcome magnitude for inferring cue-outcome contingencies in the context of this task.

Equation (3): ‘All-Normalized’ Reinforcement Learning of Cue-Values

3 $$\begin{aligned} & V \left( { SeenCue\left( t \right)} \right)_{t + 1} = V\left( { SeenCue\left( t \right) } \right)_{t} + \alpha_{m} \left( {{ }\updelta \left( {\text{t}} \right){ }} \right) \\ & V \left( { UnseenCue\left( t \right)} \right)_{t + 1} = V\left( { UnseenCue\left( t \right) } \right)_{t} - \alpha_{m} \left( {{ }\updelta \left( {\text{t}} \right){ }} \right) \\ & \delta (t) = \left( {OutcomeValence\left( t \right) {-} V\left( {SeenCue\left( t \right)} \right)_{t} } \right) \\ \end{aligned}$$

V(SeenCue(t)) = Value of the cue presented on trial t

V(UnseenCue(t)) = Value of the cue not presented on trial t

$\alpha_{m}$ = learning rate on trial t

δ(t) = Prediction Error on trial t

In Eq. (3), calculation of *δ(t)* (prediction error) relies on outcome valence as per Eq. (2) above. However, Eq. (3) implements more sophisticated RL, in that it capitalizes on the equal-and-opposite predictive significance of the two cues with respects to the probability of wins/losses, by updating not only the seen cue’s value on every trial but also the counterfactual, ‘unseen’ cue’s value (reciprocally to reflect their oppositional relationship with particular outcomes) to make full use of the symmetrical information available about each cue from the outcome of any particular trial.

### Confidence-modulation model families (Eqs. 4 – 5)

In addition to a family of models implementing no confidence-modulation of any parameters whatsoever (see Table 4), there were two families of models that did incorporate some form(s) of confidence-modulation and differed from each other in how confidence was monitored, i.e. in the trialwise confidence-update rule used:

**Table 4:** Among those 24 models which performed either kind of confidence-monitoring (i.e. either “surface” or “surprisal” based confidence updating), the effect(s) of the confidence representation on learning and/or decision-making systematically varied to define a third axis of model classification.

		How is Confidence Used to Modulate Lower-Level Parameters?			
		$\propto_{{\varvec{m}}}$	${\varvec{\beta}}_{{\varvec{m}}}$	Both﻿$\user2{\alpha m \,and\, \beta m}$with different weights (κA = / = κB)	Both﻿$\user2{\alpha m \,and\, \beta m}$with same weight, κ (κA = κB = κ)
What is the teaching signal for updating confidence?	Surprisal Eq. (3)	Basic	Basic	Basic	Basic
		Outcome-normalized	Outcome-normalized	Outcome-normalized	Outcome-normalized
		All-normalized	All-normalized	All-normalized	All-normalized
	Outcome Desirability Eq. (4)	Basic	Basic	Basic	Basic
		Outcome-normalized	Outcome-normalized	Outcome-normalized	Outcome-normalized
		All-normalized	All-normalized	All-normalized	All-normalized

Equation (4): Outcome-Desirability-based confidence update on trial t :

4 $$C _{t + 1} = C_{ t} + \gamma { }\left( {D(OOp _{t} ){ }{-}{ }C_{ t} } \right)$$

*C* = Confidence estimate (hidden state, 0 ≤ *C* ≤ 1).

γ = confidence-update rate (free parameter, 0 ≤ γ ≤ 1).

*D(O)* = Outcome Desirability (observed state, D(O) [0,1]).

Undesirable outcomes (D(O) = 0) were ‘winning only 10p’ and ‘losing £1’. Desirable outcomes (D(O) = 1) were ‘winning £1’ and ‘losing only 10p’.

Equation (5): Surprisal-based confidence update on trial t:

5 $$C _{t + 1} = C_{ t} + {\upgamma }\left\{ { \frac{{\left( {2 - \left| {{\updelta }_{ t} } \right|} \right)}}{2} - C_{ t} } \right\}$$

*C* = Confidence estimate (hidden state, 0 ≤ *C* ≤ 1).

γ = confidence-update rate at higher level (free parameter, 0 ≤ γ ≤ 1).

$$\left| {\delta t} \right| = {\text{magnitude}}\;{\text{of}}\;{\text{lower}} - {\text{level}}\; {\text{prediction}}\;{\text{error}}$$ on trial t.

### Confidence-use model families﻿ (Eqs. 6–8)

Finally, models incorporating confidence-monitoring (whether surprisal- or outcome-desirability-based) also varied in how confidence was used to modulate learning-rate and/or choice temperature. If confidence modulated both these lower-level parameters, the confidence-weighting factor for learning rate (κA) could be either the same as, or different from, the confidence-weighting factor for choice temperature (κB).

In models incorporating confidence-modulation of prediction-error driven belief-updating (i.e. κ*A* = / = 0), the effect of confidence on learning-rate differed depending on whether the outcome was contradictory or confirmatory of extant beliefs.

**For contradictory outcomes** (wherein the outcome’s valence was different from that of the current valuation of the seen cue) learning increased rapidly as a function of uncertainty, as per Eq. (6). In this way, poorly performing strategies could quickly be replaced with more adaptive ones (whereas prediction errors more likely to be noise, in that they contradicted confident beliefs, drove relatively little revision of existing value estimates).

For confirmatory outcomes, learning was instead somewhat accelerated when confidence was high (compared to when the belief being “confirmed” was more uncertain) as per Eq. (7). Thus the model instantiated a form of confirmation bias that helped to rapidly instantiate and solidify adaptive behavioural strategies:

Equation (6): Confidence-modulation of learning from contradictory outcomes

6 $$\propto_{m} = \frac{{ \propto_{0 } }}{{ 1 + \kappa_{A } C_{ t} }}$$

Equation (7): Confidence-modulation of learning from confirmatory outcomes

7 $$\propto_{m} = \frac{{ \propto_{0 } + \kappa_{A } C\left( t \right)}}{{ 1 + \kappa_{A } C\left( t \right)}}$$

$\propto_{m}$ = cue-update learning rate at lower level on trial *t.*

*C* = Confidence estimate (hidden state, 0 ≤ *C* ≤ 1).

$\kappa_{A }$ = weighting factor for confidence-modulation of learning rate (free parameter, 0 ≤ $\propto_{0 }$ ≤ 1).

$\propto_{0 }$ = cue-update learning rate when *C* = 0 (free parameter, 0 ≤ $\propto_{0 }$ ≤ 1).

The effect of confidence on *trialwise choice temperature* β*m* was specified by Eq. (8):

Equation (8): Confidence-modulation of choice temperature

8 $$\beta_{m} = \frac{{ \beta_{0 } }}{{1 + \kappa_{B } C\left( t \right)}}$$

$\beta_{m}$ = choice temperature on trial t.

*C* = Confidence estimate (hidden state, 0 ≤ *C* ≤ 1).

$\kappa_{B }$ = weight of confidence-modulation of choice temperature (free parameter, 0 ≤ $\propto_{0 }$ ≤ 1).

$\beta_{0 }$ = choice temperature when *C* = 0 (free parameter).

Thus, more confidently held beliefs were more powerful determinants of behaviour. For all models regardless of their lower-level reinforcement learning algorithm, and of whether and how confidence was monitored and used to influence lower-level parameters (including choice temperature βm, which in models that did not modulate decision-making by confidence remains fixed at βm = β0 for all trials) the estimated value of the seen cue was mapped onto the probability of choosing the ‘risky’ (vs the ‘less risky’) option by:

Equation (9): Softmax Decision-Making Rule

9 $${\text{P}}\left( {{\text{Risky}}\left( {\text{t}} \right)} \right) \, = { 1 }/ \, ({1 } + {\text{ exp}}( \, - {\text{ V}}\left( {{\text{SeenCue}}\left( {\text{t}} \right) \, / \, \beta {\text{m }}} \right)$$

All models were inverted using a variational Bayes approach^32,60^ under the Laplace approximation^61^. The logarithm of model evidences, estimated for each participant and model, were submitted to group-level analysis.

### Family-wise Bayesian model comparison

Family-wise analyses grouped models into computationally similar subsets, taking into account the fact that evidence for a particular cognitive strategy (as embodied by a single computational model) also lends evidential support to meaningfully similar models of cognition that are represented by other models within the model-space.

Families of models were defined in three different ways, by grouping models into subsets according to their computational similarity with one another on the various axes along which models could independently differ (lower-level reinforcement learning; confidence-monitoring, and confidence-modulation).

We first partitioned the model-space into families according to the reinforcement learning rule implemented at the lower level: ‘basic’ (monetary value-dependent updating of the seen cue only on each trial), ‘outcome normalized’ (updating of the seen cue only on each trial, but according to the valence rather than monetary value of the outcome), and ‘all normalized’ (outcome valence-dependent updating of both cues, equally and oppositely, on each trial).

We next partitioned the model-space according to the type of confidence-monitoring implemented at the second level: ‘none’ (no confidence monitoring), ‘surprise’ (prediction error-dependent updating of confidence), and ‘surface’ (outcome desirability-dependent updating of confidence).

Finally, we divided the model-space into five families that differed in how confidence was used to modulate lower-level parameters*:* no modulation at all (i.e. no confidence monitoring), modulation of *trialwise learning rate ****αm*** only, modulation of *trialwise choice temperature* ***βm*** only, modulation of *both* (i.e. of αm and βm )with the *same weight,* ${\varvec{k}}$, or modulation of both ***αm*** and ***βm*** with *different weights*: ${\varvec{\kappa}}_{{\user2{A }}}$ and ${\varvec{\kappa}}_{{\user2{B }}}$ respectively.

### Post-hoc exploratory analyses

Those computational parameters that significantly differed between groups (i.e. between HC1, ARMS and FEP in *Study 1*; or between HC2 and TRS in *Study 2*) were examined in relation to delusional symptoms (CAARMS NBI + UTC and/or PANSS P1). The idea here was that the free parameters defining the hierarchical reinforcement learning model of trialwise choice data may be sensitive to within-group differences in the severity of delusional beliefs, which like the beliefs that drive performance on this probabilistic reversal learning task are formed, maintained and acted upon under environmental uncertainty (noise and volatility) and subject to inter-individual variability between members of a group (e.g. delusional symptoms may be a prominent feature of some, but not other, chronic schizophrenic presentations).

## Data Availability

The NCAAPS (NSPN) data used in *Study 1* has not yet been made publicly available (as of 20/07/2023) but is already (as of this manuscript’s publication) available from the authors upon request. *Study 2*’s data is not publicly available due to this not being foreseen at the time of obtaining ethical approval, however this dataset is likewise available from the authors upon request.
